# Zero balance ultrafiltration using dialysate during nationwide bicarbonate shortage: a retrospective analysis

**DOI:** 10.1186/s13019-019-0986-8

**Published:** 2019-09-10

**Authors:** Ryan Mullane, Lance Fristoe, Nicholas W. Markin, Tara R. Brakke, Helen Mari Merritt-Genore, Aleem Siddique, Clifford D. Miles, Troy J. Plumb

**Affiliations:** 1 Department of Internal Medicine, University of Nebraska Medical Center, 983040 Nebraska Medical Center, Omaha, NE 68198-3040 USA; 2 Department of Clinical Perfusion, University of Nebraska Medical Center, Omaha, NE USA; 3 Department of Anesthesiology, University of Nebraska Medical Center, Omaha, NE USA; 4 Department of Surgery, University of Nebraska Medical Center, Omaha, NE USA

**Keywords:** Zero balance ultrafiltration, Z-BUF, Dialysate

## Abstract

**Background:**

Zero balance ultrafiltration (Z-BUF) utilizing injectable 8.4% sodium bicarbonate is utilized to treat hyperkalemia and metabolic acidosis associated with cardiopulmonary bypass (CPB). The nationwide shortage of injectable 8.4% sodium bicarbonate in 2017 created a predicament for the care of cardiac surgery patients. Given the uncertainty of availability of sodium bicarbonate solutions, our center pro-actively sought a solution to the sodium bicarbonate shortage by performing Z-BUF with dialysate (Z-BUF-D) replacement fluid for patients undergoing cardiopulmonary bypass.

**Methods:**

Single-center, retrospective observational evaluation of the first 46 patients at an academic medical center who underwent Z-BUF using dialysate over a period of 150 days with comparison of these findings to a historical group of 39 patients who underwent Z-BUF with sodium chloride (Z-BUF-S) over the preceding 150 days. The primary outcome was the change in whole blood potassium levels pre- and post-Z-BUF-D. Secondary outcomes included changes in pre- and post-Z-BUF-D serum bicarbonate levels and the amount of serum bicarbonate used in each Z-BUF cohort (Z-BUF-D and Z-BUF-S).

**Results:**

Z-BUF-D and Z-BUF-S both significantly reduced potassium levels during CPB. However, Z-BUF-D resulted in a significantly decreased need for supplemental 8.4% sodium bicarbonate administration during CPB (52 mEq ± 48 vs. 159 mEq ± 85, *P* < 0.01). There were no complications directly attributed to the Z-BUF procedure.

**Conclusion:**

Z-BUF with dialysate appears to be analternative to Z-BUF with sodium chloride with marked lower utilization of intravenous sodium bicarbonate.

## Background

Cardiopulmonary bypass (CPB) can be associated with the development of metabolic derangements, including metabolic acidosis (serum bicarbonate < 22 mEq/L) and hyperkalemia (serum potassium > 5.1 mEq/L). These derangements occur secondary to lactic acidosis and tissue hypoperfusion, hemodilution and changes in strong ion difference [1]. Hyperkalemia can occur due to hemolysis and renal dysfunction, or as a result of the high-potassium containing cardioplegia solution utilized to electrically arrest the myocardium during aortic cross-clamping [2].

Zero-balance ultrafiltration (Z-BUF) is the process by which a solution (replacement fluid) is infused into the CPB circuit and an equal volume of fluid is removed via an ultrafiltration column. This process allows small molecules to be removed from the blood, while cells and most proteins remain. Z-BUF therefore effectively allows for the removal of substances such as potassium, via convective clearance, and allows for the addition of substances, such as bicarbonate, without a net gain of fluid by the patient. Z-BUF was initially conceptualized to reduce the inflammatory mediators associated with CPB [3], though it is now commonly utilized to assist in the correction of electrolyte abnormalities that can occur during CPB.

A national shortage of sodium bicarbonate injections developed in 2017 due to delays in manufacturing [4]. Prior to the sodium bicarbonate shortage, our institution routinely used sodium chloride solutions (NaCl) [0.9% sodium chloride and 0.45% sodium chloride, usually in a 2:1 ratio] as the initial replacement fluids for Z-BUF and concurrently administered intravenous 8.4% sodium bicarbonate when needed for metabolic acidosis. With the nationwide shortage of sodium bicarbonate, the liberal use of sodium bicarbonate solution to correct metabolic acidosis was curtailed and we thus developed an alternate strategy to correct this abnormality.

Dialysate solutions contain bicarbonate with a relatively physiologic balance of electrolytes when compared to serum. Two single patient cases have been reported using Z-BUF with dialysate replacement fluid to treat both hyperkalemia and metabolic acidosis [5, 6]. With the extremely reduced supply of sodium bicarbonate, yet ongoing need to perform CPB at our facility, we elected to perform Z-BUF during CPB with dialysate solution as the replacement fluid to manage the ensuant hyperkalemia and metabolic acidosis. Herein, we describe our experience using dialysate as the replacement fluid for Z-BUF during CPB for electrolyte management in a large number of patients and show the results of a historical comparator group who underwent Z-BUF with NaCl (0.9% sodium chloride). We hypothesized that the use of dialysate as the replacement fluid for Z-BUF would show similar decreases in whole blood potassium levels and increases in serum bicarbonate levels when compared to Z-BUF with NaCl as the replacement fluid.

## Methods

Following review of the available published case reports [5, 6] regarding the use of dialysate for electrolyte correction via Z-BUF during CPB, the decision to proceed with its use was deemed appropriate by a team comprised of cardiovascular anesthesiology, cardiothoracic surgery, nephrology, perfusion and pharmacy. The utilization of Z-BUF for the specific subjects was determined at the discretion of the clinical perfusionist after consultation with the cardiovascular anesthesiologist and cardiovascular surgeon at the time of the surgical procedure. Z-BUF was usually initiated for patients who had hyperkalemia, metabolic acidosis, or both. Patients undergoing both scheduled and emergent procedures are included in this report. The “Z-BUF dialysate” group (Z-BUF-D) consisted of all patients undergoing CPB at our single institution in which Z-BUF was performed with dialysate in the first 150 days following switching to the dialysate solution. We decided to review patients within the first 150 days of the change to utilize dialysate as the replacement fluid for Z-BUF as that coincided with the greatest restrictions to sodium bicarbonate administration at our institution. Following approval from our Institutional Review Board (752–17-EP, 11/22/2017), we retrospectively collected patient demographics including age, sex, type of procedure, time spent on CPB and laboratory values from the electronic medical record. Laboratory testing that was reviewed included pre- and post-Z-BUF whole potassium levels (measured via blood gas analyzers) and pre- and post-Z-BUF bicarbonate levels obtained from venipuncture or indwelling central venous catheters). We also collected data on those patients at our single center who had undergone standard Z-BUF with NaCl (Z-BUF-S) in the 150 days prior to initiating Z-BUF-D and utilized them as a comparator group.

Patients were perfused using our institution’s standard, custom CPB circuit containing a Capiox® FX Advanced oxygenator with integrated arterial filter (Terumo Cardiovascular Group, Ann Arbor, MI) primed with 700 ml (mL) of PLASMA-LYTE A Inj. pH 7.4 (Baxter, Deerfield, IL). The Z-BUF was performed using a polysulfone fiber hemoconcentrator (Livanova, London). A 250 mL/min blood shunt was diverted off the recirculation line through the hemoconcentrator back to the venous reservoir. Dialysate solution was administered in approximately 500 mL boluses to maintain a safe venous reservoir level during the Z-BUF procedure. At our institution, Z-BUF is utilized for patients with hyperkalemia, metabolic acidosis, or those patients in whom these disorders are expected to occur because of the time or nature of the procedure. The dialysate solutions utilized were the NxSTAGE® Pure Flow™ Bicarbonate Solutions RFP-400® (2 mEq/L K^+^, 35 mEq/L HCO^−^ _3_) and RFP-402® (0 mEq/L K^+^, 35 mEq/L HCO^−^ _3_) (NxStage, Lawrence, MA) (Table 1), which are supplied as 5 l (L) bags and cost between $20 to $25 per bag. The type and volume of dialysate incorporated as replacement fluid was determined by the treatment team, with the RFP-400® solution used initially due to availability and then a switch to primarily using RFP-402® solutions (when they were obtained from NxStage®)given its lower potassium concentration. Dialysate use as replacement fluid was limited to the duration of CPB as required by the associated surgical procedure.

**Table 1:** Content of Potential Solutions Used During Z-BUF [7, 8]

Product	Bicarbonate (mEq/L)	Potassium (mEq/L)	Sodium (mEq/L)	Calcium (mEq/L)	Chloride (mEq/L)	Glucose (mg/dL)	Lactate (mmol/L)	Acetate (mmol/L)	Gluconate (mmol/L)
RFP-400®	35	2	140	3	111	100	–	–	–
RFP-402®	35	0	140	3	109	100	–	–	–
Normal saline (0.9%)	–	–	154	–	154	–	–	–	–
0.45% Sodium Chloride	–	–	77	–	77	–	–	–	–
Lactated Ringer’s	–	5.4	131	2	111	–	29	–	–
Plasma-Lyte A	–	5	140	–	98	–	–	27	23
8.4% Sodium Bicarbonate	1000	–	1000	–	–	–	–	–	–

Our analysis is a retrospective, observational comparison to a historical cohort. The primary outcome was the change in whole blood potassium levels pre- and post-Z-BUF. Secondary outcomes included changes in pre- and post-Z-BUF serum bicarbonate levels and the amount of serum bicarbonate used for Z-BUF. Normally distributed variables are presented as mean value ± standard deviation. Normal probability plots were created to determine that the data (whole blood potassium and serum bicarbonate) was normal. Student’s T test was used to compare the values of the variables between groups. *P* < 0.05 was considered an indication of a statistically significant result.

## Results

Forty-six patients underwent CPB with Z-BUF-D during the first 150 days following the change in Z-BUF protocol. Thirty-nine patients who had undergone CPB with Z-BUF-S during the 150 days prior to the sodium bicarbonate shortage were reviewed to serve as a reference group. Details regarding these subjects are summarized in Table 2. Of the Z-BUF-D subjects, 30 were male and 16 were female. The mean age of this group was 62.5 ± 12.8 years, mean duration of CPB 158 ± 77 min and mean baseline creatinine 1.6 ± 1.6 mg/dL. This cohort included four patients with end-stage renal disease (ESRD) and ten patients with chronic kidney disease stage 3–5. The volume of replacement fluid ranged from 1300 ml (mL) to 10,000 mL. The mean volume of replacement fluid used in Z-BUF-S patients was 2413.16 mL while the mean volume of dialysate used as a replacement fluid in the Z-BUF-D group was 4428.38 mL (*P* < 0.01).

**Table 2:** Characteristics of patients who underwent Z-BUF with and without dialysate as replacement fluid

Characteristics	Z-BUF-S (*n* = 39)	Z-BUF-D (*n* = 46)	*p* value
Age (years)	53.1 ± 13.6	62.5 ± 12.8	< 0.01
Female sex - number (%)	9 (23%)	16 (35%)	0.12
Pre-operative creatinine (mg/dL)	1.9 ± 2.4	1.6 ± 1.6	0.25
Pre-operative potassium (mEq/L)	4.2 ± 0.5	4.2 ± 0.4	0.34
Pre-operative bicarbonate (mEq/L)	26.2 ± 3	26.0 ± 3.3	0.38
Coronary Artery Bypass Grafting (CABG) – number (%)	15 (38%)	23 (50%)	0.31
Valve Repair – number (%)	13 (33%)	11 (24%)	
Aortic Dissection or Aneurysm Repair – number (%)	8 (21%)	8 (17%)	
Other procedure – number (%)	3 (8%)	4 (9%)	
Mortality (%)	8	7	0.39

Pre-operative potassium levels vary from pre-Z-BUF potassium levels (peak intraoperative). Patients who did not receive dialysate as a replacement fluid were from prior to sodium bicarbonate shortage. Patients who did receive dialysate as a replacement fluid were from after sodium bicarbonate shortage.

Plus-minus values are means ± standard deviation

*Z-BUF* zero balance ultrafiltration

*Z-BUF-S* zero balance ultrafiltration with sodium chloride solutions

*Z-BUF-D* zero balance ultrafiltration with dialysate solution

The mean peak intraoperative whole blood potassium level (pre-Z-BUF) was 6.4 ± 0.73 mEq/L in the Z-BUF-S group and 5.6 ± 0.68 mEq/L in the Z-BUF-D group (*P* < 0.01). The mean post-Z-BUF whole blood potassium level was 4.6 ± 0.68 mEq/L in the Z-BUF-S group and 4.3 ± 0.56 mEq/L in the Z-BUF-D group. The difference between the pre-Z-BUF and post-Z-BUF potassium was significant in both groups (*P* < 0.01). All patients undergoing Z-BUF-D had a decrease in their whole blood potassium level (with the exception of one patient, for whom information was not available as they died intra-operatively).

The mean pre-Z-BUF bicarbonate level was 26.2 ± 4.2 mEq/L in the Z-BUF-S group and 25.9 ± 3.4 mEq/L in the Z-BUF-D group (*P* = 0.31). The mean post-Z-BUF serum bicarbonate level was 22.1 ± 2.67 mEq/L in the Z-BUF-S group and 22.6 ± 2.37 mEq/L in the Z-BUF-D group. Overall, there was a decrease in the serum bicarbonate concentration following the CPB period in all patients undergoing Z-BUF. The change in serum bicarbonate was similar in the patients undergoing Z-BUF-D and Z-BUF-S (− 3.32 ± 3.96 mEq/L and − 4.18 ± 3.59 mEq/L, *P* = 0.13). The average 8.4% sodium bicarbonate used per case was significantly less in the patients undergoing Z-BUF-D (52 ± 48 mEq/patient) compared to the patients undergoing Z-BUF-S (159 ± 85 mEq/patient) (*P* < 0.01).

The post-Z-BUF serum sodium levels were similar between the Z-BUF-S (142.6 ± 3.68 mEq/L) and Z-BUF-D cohorts (141.8 ± 0.15 mEq/L) (*P* = 0.15). The post-Z-BUF serum chloride levels were also similar in the Z-BUF-S and Z-BUF-D groups (111.08 ± 4.39 mEq/L versus 109.76 ± 2.84 mEq/L) (*P* = 0.06). The post-Z-BUF pH and PaCO2 levels did not differ between the Z-BUF-S and Z-BUF-D patients.

Seven percent of the in the Z-BUF-D cohort died, all of complications unrelated to Z-BUF. There was no difference in mortality when compared to the patients undergoing Z-BUF-S (*P* = 0.39). Six patients without pre-existing ESRD required dialysis following their procedures; one patient had been on dialysis for acute kidney injury prior to their procedure and was continued on dialysis thereafter. Of the five patients who had not been on dialysis prior to their procedures, one patient required initiation of hemodialysis on post-operative day 1 due to hyperkalemia, one patient required initiation of hemodialysis on post-operative day 0 due to acidemia (due to type A lactic acidosis). One patient required hemodialysis on post-operative day 7 due to anuric renal failure likely secondary to ischemic acute tubular necrosis, one patient required hemodialysis on post-operative day 5 for volume removal and one required hemodialysis on a hospital admission within one month of their procedure.

## Discussion

Hyperkalemia and metabolic acidosis are complications that can occur during CPB. Z-BUF-S is effective at lowering potassium, but alone is inadequate to treat metabolic acidosis and often induces metabolic acidosis because of the removal of bicarbonate in the ultrafiltrate. In combination with 8.4% sodium bicarbonate, Z-BUF-S is highly effective at treating both hyperkalemia and metabolic acidosis, and prior to the sodium bicarbonate shortage, was our standard method of performing Z-BUF. In our experience, this combination resulted in the utilization of over 150 mEq of sodium bicarbonate per surgical case. In the presence of a sodium bicarbonate shortage, our institutional allotment of sodium bicarbonate could not support continuation of this protocol. Hence, in response to this shortage, we chose to use bicarbonate-containing dialysate (NxStage® RFP-400® and RFP-402®) as our Z-BUF solution.

Two prior single patient case reports suggested that Z-BUF-D could be used to correct the hyperkalemia and metabolic acidosis associated with CPB [5, 6]. Our retrospective analysis sought to further demonstrate the efficacy and feasibility of utilizing Z-BUF-D during the sodium bicarbonate shortage in a larger patient population.

Although NxStage® RFP-400® and RFP-402® solutions are not FDA-approved for intravenous use, the utilization of these solutions with Z-BUF is consistent with their use as replacement fluid during continuous renal replacement therapy, for which they are FDA-approved. Similar dialysate solutions commercially available include Duosol® (Braun, Bethlehem, PA) and Prismasate® (Baxter, Deerfield, IL). Alternative replacement fluids (Lactated Ringer’s and Plasma-Lyte A) to dialysate were considered. The composition of these crystalloids all vary considerably when compared to the dialysate solutions (Table 1). We ultimately decided that dialysate was a better option because it was available as a zero-potassium solution and it contained bicarbonate rather than acetate or lactate, both of which would require hepatic metabolism to be converted to bicarbonate, which we felt could be highly variable during CPB.

The use of dialysate as the replacement fluid in Z-BUF resulted in similar changes in the serum bicarbonate compared to patients undergoing Z-BUF with NaCl as the replacement fluids. However, the use of dialysate in Z-BUF allowed our team to use over 100 mEq less of intravenous sodium bicarbonate per patient; a critical amount during the bicarbonate shortage. Although the mean change in whole blood potassium was greater in patients undergoing Z-BUF-S, the cohort who did Z-BUF-D did meet our goals for potassium reduction. We postulate that the lower pre-Z-BUF potassium level in the Z-BUF-D cohort reflects the use of Z-BUF-D for the management of metabolic acidosis without significant hyperkalemia. Prior to the sodium bicarbonate shortage, patients with metabolic acidosis, but without hyperkalemia, would have been managed with 8.4% sodium bicarbonate without Z-BUF.

The reduction in bicarbonate use was instrumental in our institution’s ability to manage the sodium bicarbonate shortage. Although some of the reduction in bicarbonate usage may be related to greater awareness of the usage of sodium bicarbonate by the providers, we believe that the use of dialysate has played a major role in our ability to manage patients undergoing CPB. We do not believe that the use of dialysate had any adverse effects on patients. We have included patient characteristics and outcomes of patients undergoing Z-BUF-S in the 150 days prior to our conversion. The Z-BUF-S group is a sample of convenience and is included to provide the reader with an idea of our prior standard of care. Although the use of a sample of convenience limits our ability to draw direct conclusions, randomization, or even a matched cohort comparison, was not practical for this descriptive study. Despite this limitation, Z-BUF with dialysate appears to have similar outcomes to those seen with NaCl while utilizing significantly less sodium bicarbonate per surgery (52 mEq vs 159 mEq).

Our analyses has a variety of limitations. The retrospective nature of our data interpretation limits the ability to determine the reasoning by which providers determined who should receive Z-BUF as well as determine the amount of replacement fluid used. There was a lack of standardization between the Z-BUF-S and Z-BUF-D cohorts, especially in terms of procedure type, aortic cross clamp time and cardiopulmonary bypass time. The cohorts only included those patients undergoing Z-BUF during a period of 150 days prior and following the beginning of sodium bicarbonate shortage and our institution beginning to use dialysate as the replacement fluid. Despite these limitations, this retrospective analysis serves to demonstrate the feasibility of utilizing dialysate as a replacement fluid during Z-BUF.

## Conclusion

Z-BUF with dialysate replacement fluid appears to be aneffective therapy for the treatment of hyperkalemia and metabolic acidosis in patients undergoing CPB. It appears to be an effective alternative to Z-BUF with NaCl and in our experience significantly decreases the need for 8.4% sodium bicarbonate in patients undergoing CPB with Z-BUF.

## Data Availability

Available upon request.

## Abbreviations

CPB: Cardiopulmonary bypass
NaCl: Sodium chloride
Z-BUF: Zero balance ultrafiltration
